# Retraction Note: The nuclear orphan receptor Nur77 alleviates palmitate-induced fat accumulation by down-regulating G0S2 in HepG2 cells

**DOI:** 10.1038/s41598-022-09547-5

**Published:** 2022-03-29

**Authors:** Naiqian Zhao, Xiaoyan Li, Ying Feng, Jinxiang Han, Ziling Feng, Xifeng Li, Yanfang Wen

**Affiliations:** 1 Department of Gerontology, Second Hospital of Shanxi Medical University, 382 Wuyi Road, Taiyuan, 030001 Shanxi China; 2 Department of Infectious Diseases, First People’s Hospital of Jinzhong, 85 Shuncheng Street, Jinzhong, 030600 Shanxi China

Retraction of: *Scientific Reports* 10.1038/s41598-018-23141-8, published online 19 March 2018

The Editors have retracted this Article.

After publication of this Article it was brought to the Editors' attention that the data in Figure 2 (with the exception of the data for Nur 77) were previously published in Figure 2 of the authors' previous paper^1^. Data from Figure 5B were published as part of Figure 5 of the same previous publication. Additionally, multiple partial duplications between images representing different samples were identified as follows:

- in Figure 1A panel Palmitate 50 overlaps partly with the fourth image in Figure 4A;
- in Figure 1A panel Palmitate 100 overlaps partly with the fourth image in Figure 4A;
- in Figure 1A panel Palmitate 200 overlaps partly with the third image in Figure 4A;
- in Figure 1A panel Palmitate 200 overlaps partly with the second image in Figure 5B;
- in Figure 5B the second and the third image partly overlap.

The Authors are unable to provide the original data. The Editors no longer have confidence in the results and conclusions of this Article.

All Authors agree with the retraction and its wording.
